# Intracystic Papillary Carcinoma of the Breast in a Male Patient: A Case Report

**DOI:** 10.1155/2012/378157

**Published:** 2012-10-30

**Authors:** Sadık Muallaoglu, Ersin Özdemir, Lale Kutluay

**Affiliations:** ^1^Department of Medical Oncology, Faculty of Medicine, Baskent University, kışla yüreğir, 01230 Adana, Turkey; ^2^Department of General Surgery, Bayindir Hospital, 06520 Ankara, Turkey; ^3^Department of Pathology, Bayindir Hospital, 06520 Ankara, Turkey

## Abstract

Breast carcinoma is an uncommon neoplastic condition among man, accounting for not more than 1% of all breast cancers. Intracystic papillary carcinoma in man is an extremely rare condition and represents only 5–7,5% of all male breast carcinomas. Clinical and radiological manifestations of intracystic papillary carcinomas are not specific. Pathologic diagnosis can be difficult at classical histological examination and identification of myoepithelial cells layer by immunohistochemical study can be useful. Adjuvant therapy is still controversial and prognosis is excellent. We report a case of this rare histological type of breast cancer in 48-year-old male patient and review the literature.

## 1. Introduction

 Breast carcinoma in men is rare; it represents 0.6% of all breast carcinomas and less than 1% of all malignancies in men [1]. Intracystic papillary carcinoma (IPC) is a rare form of breast cancer, accounting for 0.5–1% of all breast cancers [2]. IPC in man is an extremely rare disease with only a few case reports published in the literature so far. IPC accounts for 5 to 7.5% of all male breast cancers [3]. It typically occurs at an old age with a good prognosis [4]. One study reported that 10-year survival rate for IPC is 100%; the recurrence-free survival rate is 96% and 77% at 2 and 10 years, respectively [2]. Clinical and radiologic manifestations of IPC are not specific. On ultrasonography, it can be pure cyst, a mixe image, or a solid mass. Therapeutic management of IPC is also still controversial. Endocrine therapy and radiation are used by many centers but evidence of their role in prognosis improvement is still lacking. Here, we report a case of intracystic papillary carcinoma (IPC) of breast in 48-years-old male patient and review the literature.

## 2. Case Report

**Figure 1:**
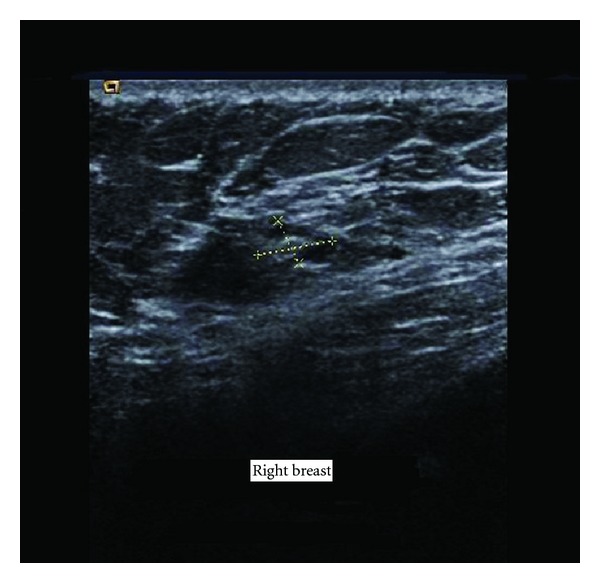
Ultrasonography showed heterogeneous mass with cystic and solid components, measuring 19 × 10 × 11 mm.

A 48-year-old man presented in General Surgical Department of our hospital with painful round swelling in the right breast, since 1-month duration. He had noticed a rapid increase in size since 10 days. There was no history of systemic disease, trauma, or family cancer history. On physical examination, there was no gynaecomastia. A 2 × 3 cm well-circumscribed, firm, painful mobile mass was noted in periareolar region. Nipple was not retracted. Bilateral axillary lymph nodes were not palpable. Left breast was normal. On sonography the mass was heterogeneous with solid and cystic components, measuring 19 × 10 × 11 mm (Figure 1). These finding suggested a benign tumor and a surgical excision was performed.

**Figure 2:**
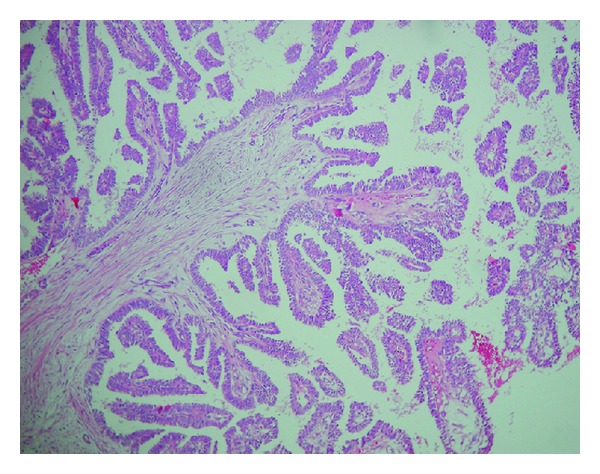
Low power magnification of papillary structures showing multilayer cells covering a fibrovascular stalk (H. E. ×100).

**Figure 3:**
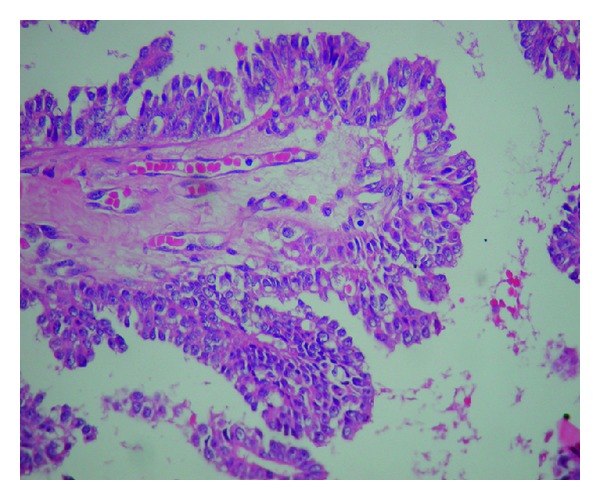
Higher magnification of one of the papillary structures (H. E. ×400).

**Figure 4:**
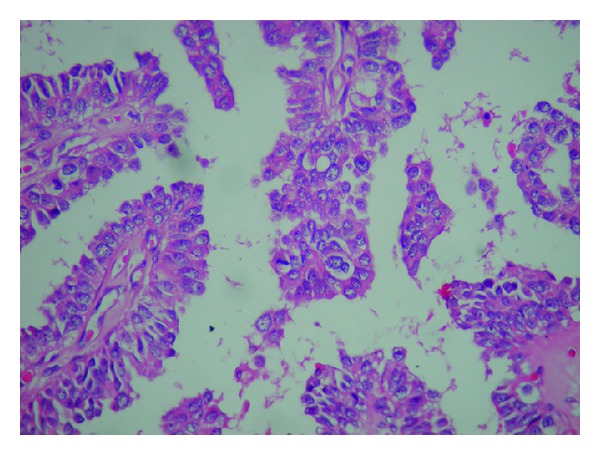
Multiple papillary structures with one in the center showing a mitotic figure (H. E. ×400).

**Figure 5:**
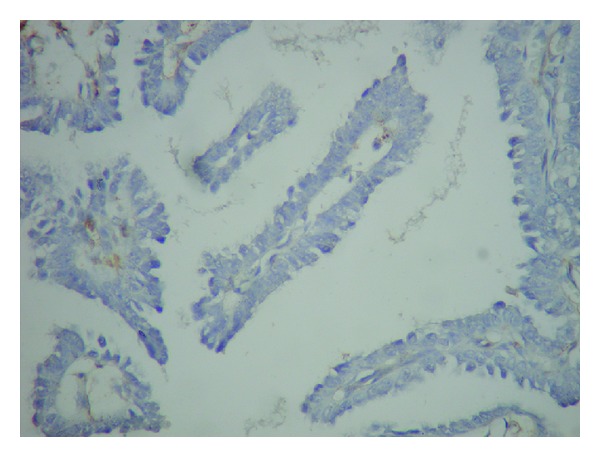
Immunohistochemical SMA stain showing the absence of myoepithelial cell layer in papillary structures (×400).

Operation material was sent for frozen section. Material received was 3 × 3 × 2, 5 cm and was lipomatous. The cut section showed 2 cystic structures measuring 0,4 cm and 0,5 cm. Both were filled with serosanguinous material. Frozen section diagnosis was “papillary neoplasm” and final diagnosis was left to permanent sections. Permanent sections revealed a dilated duct with papillary structures protruding into the lumen (Figures 2 and 3). These papillary structures had fine fibrovascular cores and were covered with multilayered cells showing prominent nuclear pleomorphism and brisk mitotic activity (Figure 4). Immunohistochemical smooth muscle actin (SMA) stain showed the absence of myoepithelial layer in the papillary structures. Final diagnosis was “intraductal papillary carcinoma” (Figure 5).

Our patient was free of disease about two years after the excision.

## 3. Discussion

Intracystic breast carcinoma is rare in females and exceedingly rare in males with a handful of case reports in the literature. The IPC is more frequently found among postmenopausal woman with an average age between 55 and 67 years old [5]. Many cases were also described in the male population in the literature, and it is the second men's breast cancer [6, 7]. IPC in man is usually reported among those of an older age group (67 to 84 years) [3]; however, in our patient, IPC developed significantly in younger age.

Histologically, IPC is divided into 3 subgroups. Pure IPC, IPC associated with DCIS, and IPC associated with invasive carcinoma. The majority of patients with IPC have associated ductal carcinoma in-situ (DCIS), or invasive carcinoma, or both and the treatment strategies differ on the basis of this associated pathology [8]. Hill and Yeh, using myoepithelial cell staining, suggest a spectrum of progression from in situ disease to invasive disease, signifying that what appears to be DCIS on histology may potentially cause distant metastases [9]. The lack of an intact basal myoepitel cell layer can be identified by calponin, smooth-muscle myosin heavy chain (SMM-HC) cytoplasmic stain, and by p63 nuclear stains. This “gold standard” method has a relatively high sensitivity and denotes the invasiveness of the tumour cells in malignant papillary breast lesions [9].

The diagnosis of IPC of the male breast should be made carefully. Triple assessment is essential (clinical examination and radiological and histological assessment). IPC tends to be well defined on mammography; an irregular margin suggests the presence of invasion [10]. Ultrasonography typically reveals a hypoechoic area (representing the cyst) with soft tissue echoes projecting from wall of the cyst (intracystic tumour) [11]. Contrast-enhanced MRI may show marked enhancement of cyst walls, septations, and mural nodules [12].

Fine-needle aspiration cytology and core biopsy are usually performed; however, the false negative results with cytology are relatively frequent [13]. Therefore, excisional biopsy should be carried out in all cystic lesions of the male breast which are suspicious on any of the above diagnostic modalities. The surgical excision allows the pathologist to classify the papillary lesion by classical histological examination and especially with immunohistochemical study and to research invasion or DCIS in surroundings breast tissues, present in majority of cases [14].

There are no evidence-based guidelines for treatment of IPC. There is no randomized controlled trial comparing breast conserving surgery to mastectomy. However, many case reports and retrospective studies showed excellent prognosis with conservative surgery without axillary dissection in IPC not associated to DCIS or microinvasion lesions [2, 8, 15]. Sentinel node biopsy may be an excellent alternative to full axillary dissection in patients with IPC and associated invasive carcinoma [2]. Cutuli et al. were reported 31 male cases of ductal carcinoma in situ of the breast. Six of the 31 cases underwent lumpectomy and 25 mastectomy. All cases had negative nodes. Relapse-free survival was 83% at 10 years, since four cases had local relapse [16]. Lefkowitz et al. [17] reported 77 female cases invasive or non-invasive IPC of the breast. These cases had been treated with excisional biopsy or mastectomy and had a 10-year disease-free survival rate of 91%.

There is also, lack of evidence about the role of adjuvant therapy. Fayanju et al. reviewed the management of 45 patients with IPC in order to determine factors associated with the use of adjuvant therapy [8]. In this study, authors concluded that the most important factor determining the use of radiation and endocrine therapies is associated pathology (DCIS or microinvasion) and patients with pure IPC were less likely to undergo radiation and endocrine therapies. In their retrospective review of the treatment and outcome of 40 patients with IPC, Solorzano et al. observed that 30% of the patients had received adjuvant radiotherapy. The addition of radiation to the treatment of patients did not change the incidence of local recurrence or the likelihood of death compared to those who did not receive radiation [2]. Grabowski et al. confirmed that the addition of hormonal treatment does not appear to have impacted the outcome [7].

In Conclusion, IPC of male breast is an extremely rare entity with favorable prognosis. Clinical and radiologic manifestations of IPC are not specific. On ultrasonography, it can be a pure cyst, a mixed image, or a solid mass. Pathologic diagnosis can be difficult at classical histological examination and identification of myoepithelical cells layer by immunohistochemical study can be useful. The mainstay of treatment is surgical resection, with adjuvant therapy if associated to DCIS or invasive carcinoma.
